# Role of Spindle Oscillations across Lifespan in Health and Disease

**DOI:** 10.1155/2016/8103439

**Published:** 2016-08-18

**Authors:** Julie Seibt, Igor Timofeev, Julie Carrier, Adrien Peyrache

**Affiliations:** ^1^Department of Biochemical Sciences, Faculty of Health and Medical Sciences, University of Surrey, Guildford GU2 7XH, UK; ^2^Department of Psychiatry and Neuroscience, Université Laval, Quebec City, QC, Canada G1J 2G3; ^3^Centre de Recherche de l'Institut Universitaire en Santé Mentale de Québec (CRIUSMQ), Université Laval, Quebec City, QC, Canada G1J 2G3; ^4^Department of Psychology, Université de Montréal, Montréal, QC, Canada H2V 2S9; ^5^Center for Advanced Research in Sleep Medicine, Hôpital du Sacré-Cœur de Montréal, Montréal, QC, Canada H4J 1C5; ^6^Centre de Recherche de l'Institut Universitaire de Gériatrie de Montréal, Montréal, QC, Canada H3W 1W5; ^7^Department of Neurology and Neurosurgery, Montreal Neurological Institute, McGill University, Montreal, QC, Canada H3A 2B4

Among the most exciting open questions in neuroscience is why and how sleep benefits our cognitive functions. Although the “why” remains a controversial topic, there is increasing evidence that a partial answer to the “how” may be found in spindle oscillations (~8–16 Hz). Spindle oscillations are central in a large variety of brain functions including somatosensory development, thalamocortical sensory gating, synaptic plasticity, and memory consolidation. Although the evidence of a link between spindles and brain plasticity is compelling, most results are still based on correlations and our knowledge of the underlying mechanisms at the basis of this relationship remains elusive. The contributing articles in this issue cover essential concepts and hypothesis underlying the physiology of spindles generation and how they contribute to cognitive functions and dysfunctions across lifespan.

Spindles are isolated oscillatory events detected across the cortex by large-scale electrophysiological measures (e.g., electroencephalography [EEG] and local field potential [LFP]). Together with slow waves (<4 Hz), spindles form the ubiquitous hallmark of NREM sleep and oscillate at frequencies between 7 and 16 Hz in animals and 11 and 16 Hz in humans. Depending on the species and the brain region examined, spindles characteristics (e.g., frequency, duration, and amplitude) can vary significantly. Although spindles are one of the most studied sleep oscillations, their intrinsic variability has been an obstacle to define standardized detection criteria and many studies still use human visual scoring for their characterization. This issue is addressed by D. C. Wallant et al. who provide a systematic review of what is known about spindles' physical parameters, focusing mainly on adult humans, and how their core characteristics can be used to develop useful automatic spindle detection methods (ASDM). ASDM are important as they provide an important starting point for reproducible data analysis and comparisons between populations (e.g., healthy versus pathologic).

The fact that spindles are heterogeneous is an intriguing observation and likely contributed to the idea that spindles are linked to individual cognitive traits, such as IQ, for example. Indeed, spindles have strong inter- and intraindividual differences, vary considerably across lifespan, and parallel the rise and decline of cognitive abilities. Although many factors certainly contribute to this heterogeneity, one potential source may directly result from the physiology of spindles generation. Spindles events are generated by the thalamocortical loop with a leading role of the thalamic reticular nucleus. The anatomical substrate and physiological processes at the basis of spindles generation are here reviewed by G. Piantoni et al. who then propose that the subdivision of the thalamocortical projections into core and matrix pathways may explain a substantial part of spindles' variability. It follows that if spindles reflect the topography and activity of thalamocortical projections, the main pathway mediating sensory processing, it is expected that spindles will also change with early development, a period when those projections are still maturing and refining. I. J. McClain et al. directly address this topic in a study where they measured changes in spindles characteristics using ASDM (and associated EEG sigma power (~11–16 Hz)) in a cohort of children between 2 and 5 years of age and showed significant variations in spindle features, likely reflecting maturation of network (e.g., thalamocortical) connectivity.

The function of sleep spindles remains elusive but evidence from the developmental and memory fields points at a fundamental role for those oscillations in brain development and plasticity. Spindle-like oscillations in humans (i.e., “Delta brush”) and animals (i.e., “spindle-burst”) are the predominant spontaneous EEG activity detected around birth. Compared to adult spindles, spindle-like oscillations cover a larger frequency range (e.g., ~8–25 Hz) and can occur across the entire sleep-wake cycle. Two reviews in this issue (J.-W. Yang et al. and C. Lindemann et al.) describe in detail the characteristics and physiology of spindle-like events at the very early stages of development in humans and rodents. The papers also present evidence that, unlike adult spindles, the topography of early spindles is much more restricted and is closely linked to the emergence and refinement of our somatosensory cortical maps. Importantly, in support for a role of those oscillations in early cortical network maturation, spindle-like events are almost exclusively triggered by spontaneous or evoked peripheral sensory activity. This includes proprioceptive feedback from muscles twitches during REM sleep, a phenomenon that is largely predominant during early development and contributes to the development of the sensorimotor circuits, as reviewed here in A. Tiriac and M. S. Blumberg's paper.

During adulthood, the role of spindles has been mainly studied in the context of cognitive functions, in particular memory formation in humans. In this issue, D. Ulrich provides an overview of the numerous studies that link changes in EEG spindles with performances in different learning tasks in humans and animals. While manipulation of spindles (e.g., transcranial magnetic stimulation, pharmacology, and optogenetic tools) can affect behavioral output and therefore support their influence on memory formation, the vast majority of data on this subject remains correlative. In fact, the actual impact of spindles on brain plasticity mechanisms during development or adulthood is still hypothetical and is based on reductionist (e.g.,* in vitro*) and computational models. However, D. Ulrich and C. Lindemann et al. discuss this aspect in their papers and propose molecular mechanisms and pathways activated during spindles across lifespan.

The forms and functions of spindles across lifespan share similarities but exhibit also differences, as detailed in a comprehensive review by B. C. Clawson et al. The question therefore remains whether those oscillations represent the same phenomenon or whether they are separate events regulating separate physiological and/or plasticity mechanisms. C. Lindemann et al. suggest that the switch in plasticity mechanisms observed during development, related to massive changes in the type and the number of brain connections, can explain the adaptation of spindles' function across lifespan. In their paper, A. Tiriac and M. S. Blumberg proposed that twitch-related spindle-bursts during immature REM (i.e., active) sleep actually persist into adulthood where they may achieve a similar function. More sensitive methods of detection will be needed to clarify this question.

Taken together, the most robust findings seem to indicate that spindles characteristics mirror the rise and decline of cognitive aptitudes and reflect the dramatic changes in the structure and function of neuronal connections across lifespan, as discussed by B. C. Clawson et al. in their paper. But whether the changes in spindles' features are in any way causal to plasticity processes remains an open question.

Sleep spindles are believed to play a crucial role in brain plasticity and memory and in protecting sleep from disturbing stimuli. Furthermore, studies seem to indicate that spindles are linked to cerebral and cognitive integrity in several pathological conditions. Not surprisingly, many studies evaluated spindles or spectral power in the sigma frequency band (11–16 Hz) to better identify brain mechanisms and functional consequences of specific sleep disorders. In this special issue, O. M. Weiner and T. T. Dang-Vu present a systematic review of 27 studies comparing spindle activity between individuals with and without sleep disorders (e.g., hypersomnia, sleep disorder breathing, insomnia, sleep-related movement disorder, and parasomnia). Studies were highly inconsistent in terms of both methodology and results which prevents clear conclusions and highlights the crucial need for future research using more stringent criteria. Despite the fact that insomnia is the most prevalent sleep disorder, O. M. Weiner and T. T. Dang-Vu report that whereas 8 studies compared sigma activity between individuals with and without insomnia, only one study compared visually detected spindles. In this issue, M.-P. Normand et al. evaluated automatically detected spindles in individuals with paradoxical insomnia or psychological insomnia and in good sleepers. They also tested the hypothesis that spindles characteristics are linked to the perception of sleep. Results indicated that most spindles characteristics did not significantly differ between the three groups of participants but that sleep spindles during stage 2 sleep were shorter in individuals with paradoxical insomnia than in good sleepers.

A number of studies indicate that sleep spindle characteristics predict cognitive status in neurological conditions across the lifespan. In this special issue, R. Gruber and M. S. Wise present a thorough literature review on sleep spindle characteristics in typically developing children and in children with neurodevelopmental disorders. They also discuss the relationship between spindles and cognition in children with and without neurodevelopment disorder. They describe the alterations in spindles in children with intellectual disability, autism spectrum disorder, and attention deficit/hyperactivity disorder. The authors also comment on the fact that most studies used small sample sizes and heterogeneous populations and that the field crucially needs replication. At the other end of life, research results also support the hypothesis that neurodegenerative diseases such as Alzheimer and Parkinson diseases show modifications in spindles characteristics. In this special issue, M. Gorgoni et al. compared automatically detected spindles between patients with Alzheimer disease (AD), individuals with mild cognitive impairment (MCI), and controlled participants. Results indicated that both individuals with MCI and AD showed lower parietal fast spindle density compared to controlled participants. Not surprisingly, knowing these group differences, parietal fast spindle density was significantly associated with Mini-Mental State Examination scores. Future studies will have to determine whether spindles may predict AD conversion in MCI participants.

Despite more than 80 years of observation and investigation, the role of spindles in brain functions remains controversial. Unravelling biomarkers of cognitive processes, such as spindles, will take us one step closer to the understanding of many brain disorders. In a series of reviews and reports, this issue presents a comprehensive overview of the current knowledge on the generation and development of spindles as well as their correlation with cognitive aptitudes in normal and neuropathological conditions. Needless to say, much work still needs to be done.

The development of large-scale imaging and high channel count electrophysiology together with optogenetic tools that allow precise manipulation of neuronal circuits provides promising perspectives in the discovery of the key properties and functions of spindle oscillations in animal models. In parallel, the recent advances in electrophysiology (e.g., single unit activity in epileptic patients) and imaging in humans will greatly contribute to the establishment of new avenues to investigate the relationship between spindles and cognitive functions. Finally, several national and international initiatives now aim to make large datasets of animal and human brain signals publicly available, together with detailed description of age, sex, and genetic background among other information. This will allow comparison and quantification of brain wave patterns at an unprecedented scale.

Detection and quantification of spindle oscillations still need to be improved, standardized, and validated. Given the importance of spindles for cognition in both humans and animals, there is no doubt that this EEG hallmark will become an important biomarker for routine health checks. Monitoring and manipulating spindle oscillations may provide future preventive cognitive training and reeducation after, for example, brain strokes.

*Julie Seibt* *Julie Seibt* *Igor Timofeev* *Igor Timofeev* *Julie Carrier* *Julie Carrier* *Adrien Peyrache* *Adrien Peyrache*

